# How much do elders with chronic conditions know about their medications?

**DOI:** 10.1186/1471-2318-13-59

**Published:** 2013-06-13

**Authors:** Frank Wan-kin Chan, Fiona Yan-yan Wong, Wing Yee So, Kenny Kung, Carmen Ka-man Wong

**Affiliations:** 1 The Jockey Club School of Public Health and Primary Care, The Chinese University of Hong Kong, Prince of Wales Hospital, Shatin, Hong Kong; 2 Department of Medicine and Therapeutics, Prince of Wales Hospital, Shatin, Hong Kong; 3 Lek Yuen GOPC, Department of Family Medicine, New Territories East Cluster, Hospital Authority, Shatin, Hong Kong

**Keywords:** Medication knowledge, Side-effects, Chronic illness, Elderly

## Abstract

**Background:**

Patients with chronic diseases often undertake multiple medication regimes to manage their condition, prevent complications and to maintain their quality of life. A patient’s medication knowledge has been defined as the awareness of drug name, purpose, administration schedule, adverse effects or side-effects and special administration instructions. Poor medication knowledge can have a negative impact on medication adherence and patient safety and, in increasing the use of medical resources. The objective of the study is to assess the medication knowledge of elderly patients with chronic disease conditions and the factors affecting this knowledge.

**Methods:**

A cross-sectional survey was conducted in patients aged ≥60 with chronic disease conditions or their caregivers were recruited from two general outpatient clinics and two medical outpatient clinics in the public sector. Participants were approached by trained interviewers to complete a semi-structured questionnaire to assess their understanding of the instructions and information relating to their regular medications, which included medication name, regimen, purpose and common side-effects and precautions.

**Results:**

A total of 412 patients were recruited with the mean age of 72.86 ± 7.70. Of those, 221 (54.2%) were male and 226 (55.4%) were of primary school educational level or below. The mean number of medications taken per patient was 3.75 ± 1.93. Overall, 52.7% of patients felt that healthcare staff or clinic pharmacists had very clearly explained the administration instruction of the prescribed medications whilst 47.9% had very clear explanations of drug purpose but only 11.4% felt they had very clear explanations of side-effects. 396 patients (96.1%) failed to recall any side effects or precautions of each of their prescribed medications, although 232 patients (58.4%) would consult a doctor if they encountered problems with their medications. Logistic regression analysis showed that for every additional medication prescribed, the likeliness of patients to recall side-effects of all the medications prescribed was significantly lowered by 35% (OR = 0.65; 95% CI = 0.44-0.94; P = 0.023). In addition, those who finished secondary school or higher education were likely to possess more knowledge of side-effects (OR = 9.88; 95% CI = 2.11-46.25; P = 0.004).

**Conclusions:**

Patients who take medications for their chronic diseases generally lack knowledge on side-effects of their medications which could potentially affect medication compliance and medication safety.

## Background

According to the World Health Organization’s multidimensional adherence model (MAM), medication adherence is shown to be multifactorial [1]. The five dimensions of MAM include socioeconomic factors, health care system-related factors, condition-related factors, treatment-related factors, and patient-related factors; of which knowledge of medication is an important component of appropriate medication use. Patients with chronic diseases often undertake multiple medication regimes to manage their conditions, prevent complications and to maintain their quality of life. Many patients with chronic disease conditions are elderly and may struggle in following medication instructions, such as taking the prescribed dosage and following the administration schedule.

A patient’s medication knowledge has been defined as the awareness of the drug name, purpose, administration schedule, adverse effects or side-effects, or special administration instructions [2]. Patients who need to take multiple medications are usually deficient in medication knowledge [3] especially the elderly. A study of patients aged 65 years of age or above found that 62% showed understanding of their medication regimens but those with the most complex regimens had difficulty with naming and explaining the purpose of their medications [4]. Another study found that 75% of patients with chronic obstructive pulmonary disease and heart failure were adherent to their prescribed medicines but their medication knowledge was low [5]. For ambulatory cardiovascular patients, a study showed that they had the highest scores for knowledge of the drug regimen and of the drug purpose, however, only a very small group could accurately provide side-effect information about the drugs they were taking [2].

Poor medication knowledge can have a negative impact on medication adherence which may result in increased use of medical resources, such as physician visits, laboratory tests, unnecessary treatments, emergency department visits, hospital admissions and treatment failure [6]. A study on patients with congestive heart failure found that those with better knowledge of their prescribed dose had a significant reduced number of cardiovascular-related emergency room visits [7]. In addition, the risk of hospitalization doubled for patients with diabetes mellitus, hypercholesterolemia, hypertension or congestive heart failure who were non-adherent to the prescribed therapies [8]. Poor medication knowledge can also lead to medication wastage. Patient-related factors leading to unused and discarded medication remains unknown, however there are huge cost implications. In the UK, it has been found that unused prescription medicines costs the NHS at least £300 million a year in 2009, of which £90 million worth of unused prescription medicines were stored in people’s homes at any one time [9]. In Hong Kong, a study found that 30% of chronically ill patients did not take medicine according to doctors’ instructions which meant that 16% of medicine (HK$40.85 in prescription fee terms) dispensed every month is wasted [10].

In this study, we assessed patient’s medication knowledge, specifically the number of medication prescribed, purpose, route, dosage, frequency and side-effects / precautions of the medications of patients aged 60 or older with chronic conditions. We also assessed the factors affecting their knowledge. We hypothesized that as patients were prescribed more medications, they would have significantly less medication knowledge, in particular in recalling side effects of each medication. The focus of our study were patients of age 60 years or older with chronic disease conditions as they were more likely to be taking multiple medications for the long term. Increased complexity of the medication schedule and longer duration of the medication regimen represented risk factors to adherence [11].

## Methods

### Setting and subjects

This was a cross-sectional survey, conducted during November 2011 to May 2012. Patients aged 60 or older with chronic conditions or their caregivers were recruited from two general outpatient clinics and two medical outpatient clinics. Patients who spoke a non-Cantonese dialect or a different language, had conditions that prevented effective communication (for example, patients who are deaf, mute, or have dementia or other psychological disorders) were excluded from the study. However, their accompanying caregivers were approached and invited to participate in the study if they were able to communicate effectively. If the caregivers were also unable to communicate effectively, the cases were excluded.

Written informed consent was obtained before initiating the interview. This study was approved by the Joint Chinese University of Hong Kong and New Territories East Cluster Clinical Research and was performed in accordance with the World Medical Association’s Declaration of Helsinki.

### Interview procedures

All consecutive patients or their caregivers were approached by trained interviewers after receiving their prescribed medications from the clinic pharmacy and were informed about the rationale and procedure of the study. If they agreed to participate, they were screened for their eligibility for participation such as the age and presence of chronic conditions. Written informed consent was obtained before entering the study. All patients or caregivers were interviewed by trained interviewers using a semi-structured questionnaire to assess their understanding of instructions and information relating to their on hand dispensed medication.

### Measuring instrument

The study questionnaire was developed based on the “Medicine Knowledge Assessment Form” developed by the American Society on Aging and American Society of Consultant Pharmacists Foundation [6]. Patients or caregivers were first asked whether they had received and understood the instructions and information given by the staff of the pharmacy. Knowledge of the number of medications prescribed by the pharmacy, the purpose of the medications, the regimen, including dosage, frequency and administration route were assessed. Patients or caregivers were also asked to name at least one side-effect or precaution of all their medications prescribed based on their own knowledge of the drug not on the basis of their own experiences. Self-reported clarity of the explanations received from healthcare staff relating to medication instructions and information was sought, as well as their preferences of healthcare professionals in seeking help for potential medication problems, along with socio-demographic information were also obtained. To ensure subject recruitment procedures and the validity and reliability of the measuring instrument, a pilot study of 20 patients with chronic conditions or their caregivers were conducted before administering the main study.

### Data analysis

The correctness of each of the answers relating to medication knowledge was examined and were categorized them as “Correct”, “Incorrect” or “Don’t know” for data analysis. The data were analyzed using SPSS (Version 16.0). Descriptive statistics on the knowledge of number of medication prescribed, medication purpose, regimen, and common side-effects /precautions were reported. Potential determinants of the knowledge of medications were explored using both univariate and multivariate logistic regression models. In the logistic regression models, knowledge of the side effects was categorised into “yes” and “no” where “yes” category included all subjects who correctly knew side-effects of each of their medications. Knowledge of medication purpose and regimen were categorised similarly. Multivariate logistic regression models were adjusted for age and gender.

## Results

We approached 787 eligible subjects. Three hundred forty subjects refused to participate and 35 were not able to complete the survey. Four hundred and twelve subjects completed the survey with a response rate of 52.4%. The mean age of the patients was 72.86 ± 7.70 years and 221 (54.2%) were male (Table 1). One hundred eighty two (44.7%) attained to secondary school level or above. Most of them (90.2%) were not working, and 21.8% received no income. Three hundred thirty six (81.6%) lived with their families or relatives.

**Table 1:** Patients characteristics

	**N = 412**
	**n (%)**
**Gender**	
M	221 (54.2%)
F	187 (45.8%)
*Missing*	4
**Age (yr)**	Mean: 72.86 ± 7.70
**Education**	
No formal education	73 (17.9%)
Kindergarten	3 (0.7%)
Primary school	150 (36.8%)
Secondary school	132 (32.4%)
University or above	50 (12.3%)
*Missing*	4
**Marital status**	
Single	11 (2.7%)
Married / Cohabit	312 (76.7%)
Widowed / Divorced	84 (20.6%)
*Missing*	5
**Employment**	
Not working	367 (90.2%)
Full-time	20 (4.9%)
Part-time	20 (4.9%)
*Missing*	5
**Government allowance**	
Old age allowance	244 (61.2%)
Disability allowance	18 (4.5%)
CSSA^*#*^	47 (11.8%)
Others	11 (2.8%)
None received	114 (28.6%)
*Missing*	13
**Monthly family income ($)**	
No income	89 (21.8%)
1–5,000	81 (19.9%)
5,001-10,000	39 (9.6%)
10,001-20,000	41 (10.0%)
20,001-30,000	19 (4.6%)
>30,000	17 (4.2%)
Don't know / Unwilling to answer	122 (19.9%)
*Missing*	4
**Living with**	
Family / Relatives	336 (81.6%)
Old age home	9 (2.2%)
Others	2 (0.5%)
Live alone	62 (15.2%)
*Missing*	3

^*#*^*CSSA: Comprehensive Social Security Assistance for eligible low-income individuals.*

Table 2 showed the average number of medications prescribed was 3.75 ± 1.93. Most subjects thought that the healthcare staff or clinic pharmacists, at least to a certain extent, explained the purpose (70.1%) and the administration instruction (72.2%) of the prescribed medications, but 73.0% claimed that they did not receive any information on side-effects or adverse effects (Table 3). When they encountered problems relating to their medication, 232 (58.4%) would consult a doctor whilst 63 (15.9%) would not take any action.

**Table 2:** Number of medications prescribed

	**N = 412**	
	**n (%)**	
**Number of medications prescribed**		
1	40 (9.7%)	Mean = 3.75 ± 1.93
2	75 (18.2%)	Median = 3.00
3	104 (25.2%)	
4	66 (16.0%)	
5	50 (12.1%)	
6-11	77 (18.7%)	

**Table 3:** Practices related to medication management

**Did the healthcare staff / clinic pharmacists explained to you:**	**Very clear**	**To a certain extent**	**Did not explain**	**No need**
Drug purpose (n = 409)	196 (47.9%)	91 (22.2%)	104 (25.2%)	18 (4.4%)
Administration instruction (n = 410)	216 (52.7%)	80 (19.5%)	81 (19.8%)	33 (8.0%)
Side-effects / Adverse effects (n = 408)	47 (11.4%)	38 (9.3%)	298 (73.0%)	25 (6.1%)
**Patient preferences in drug problems (can choose more than one option) ** **(N = 412)**	n(%)			
Consult doctor	232 (58.4%)			
Consult nurse	18 (4.5%)			
Consult clinic pharmacist	39 (9.8%)			
Consult families / friends	24 (6.0%)			
Book / internet	9 (2.3%)			
Others	41 (10.3%)			
No action	63 (15.9%)			
*Missing*	15			
	**Yes**		**No**	
**Recalling side-effect / precautions of all medications prescribed (N = 412)**	16 (3.9%)		396 (96.1%)	

The subjects’ understanding of each of their prescribed medications was also assessed. We found that most of them could answer correctly the number (83.3%), the purpose (76.2%), the route (99.0%), the dosage (80.7%) and the frequency (74.1%) of all their prescribed medications. However, only 16 (3.9%) could state the side-effects for all their prescribed medications correctly (Table 3). Among the 396 (96.1%) who either did not know or incorrectly stated the side-effect or precautions, 111 (27.0%) did not know side-effects of some medications, 109 (26.5%) did not know side-effects of all medications, whilst 176 (42.7%) incorrectly stated side-effects of all medications (Table 4).

**Table 4:** Patients responded “Don’t know” or “Incorrectly state” the side-effects of medications prescribed

	**N = 396**
**“Don't know”**	
% of medications which patients responded “Don’t know”	
10%-33%	52 (12.6%)
34%-66%	27 (6.6%)
67%-99%	32 (7.8%)
100%	109 (26.5%)
**“Incorrectly state” the side-effects of all medications**	176 (42.7%)

Subjects who “did not know” or “incorrectly stated” side-effects were grouped as “unable to recall” side-effects. Univariate logistic regression showed that, for every additional prescribed medication, the likeliness of recalling side-effects of all prescribed medications was significantly lowered by 35% (OR = 0.65; 95% CI = 0.64-0.93; P = 0.019). For every one year increase in age, the likeliness of recalling side-effects of all medications was significantly lowered by 8% (OR = 0.92; 95% CI = 0.85-0.99; P = 0.020). Those who attained secondary school educational level and above, were significantly more likely to recall side-effects of all prescribed medications (OR = 9.33; 95% CI = 2.09-41.62; P = 0.003). Multivariate logistic regression analyses showed similar results (Table 5).

**Table 5:** Factors affecting recalling side-effects of all medications prescribed using logistic regression analysis

	**Recognizing side-effects of all medications prescribed**	
	**Crude OR (95% CI)**	**Adjusted OR (95% CI)**
**Number of medications prescribed (for every additional medication)**	0.65 (0.64-0.93)	0.65 (0.44-0.94)
**Age (for every additional year)**	0.92 (0.85-0.99)	0.94 (0.87-1.02)
**Education level**		
Secondary school or above	9.33 (2.09-41.62)	9.88 (2.11-46.25)
Primary school or below	1.0 (reference)	1.0 (reference)

## Discussion

Overall, we found that participants knew the number, purpose, route, dosage and frequency of all prescribed medications well. However, they were found to have poor knowledge of the side effects of their medication. Our finding is comparable with other international studies. A Danish study of the elderly at home of age 75 years or older showed that whilst 60% knew the indication for their treatment; only 6% could report potential risks, side-effects or interactions [12]. Another study conducted in Sweden demonstrated 84% of the frail elderly did not have any knowledge about possible adverse effects for any of their prescribed medication. Only a few were aware of side effects for one or two medicine and only one patient was able to report risks or adverse effects for all their medication [13]. A study of ambulatory cardiovascular patients on medication knowledge found that subjects recorded the highest scores for knowledge relating to drug purpose and regimen, with only a very small group able to provide information about side effects accurately [2].

Knowledge of adverse effects is a very important component of medication knowledge. Patients who are not familiar with side-effects of their medication have a higher risk of serious complications. Non-steroidal anti-inflammatory drugs, medicine commonly used to treat pain in osteoarthritis, can lead to gastrointestinal bleeding; diabetic patients taking sulfonylureas can suffer hypoglycemia if taken without sufficient food intake. Some side-effects can lead to serious complications which can lead to hospitalization and are potentially life-threatening. It is very important that patients know when to seek help when they are taking medications, therefore, information on the adverse effects of medication, in particular common side effects, should be clearly explained to patients.

In this study, although 60% of the participants would consult a doctor when they encountered medication problems, 73% of the participants reported that they did not receive any information on side-effects during the consultation, prescribing and dispensing procedure. A study on the doctor-patient consultation resulting in the prescription of cardiovascular medication found that the physician was more likely to discuss the general information (86%) and drug regimen (68%), and less likely to discuss medication side-effects (26%) [2]. In our study, patients appear to be most uninformed about side-effects of their medication. The high percentage of ‘don’t know’ responses is likely the result of a lack of information provision by the physician and other healthcare staff, such as nurse and clinic pharmacist. Possible reasons for the lack of information given regarding side-effects may due to the fear of unduly alarming patients, prioritizing information about the purpose and administration schedule of the medication, and/or lack of time.

In the routine primary care clinic or specialist clinic, there may be insufficient time for the doctor to discuss with the patient every commonly encountered side-effects of all their medications. In addition, patient’s recall of information may be affected if the consultation is loaded with other additional information or if the consultation is rushed or patient’s concentration is set on other agendas. Currently, dedicated pharmacist clinics have been set up in many specialist outpatient clinics to manage certain groups of patients, such as patients on warfarin or diabetic patients. Drug information including side-effects and possible drug interactions would be provided by a pharmacist or a diabetic nurse. However, this valuable service has not been generalized to include all other medications because of limited resources. In addition, pharmacists can often only provide ‘limited and important’ information to patients due to the huge workload demands in public pharmacies.

Nevertheless, information on side-effect of medications is essential. Suggestions to improve patient education include the provision of drug information pamphlets to patients. The pamphlet can contain practical information such as medication regimen, managing multiple dosage schedules and common side-effects of all the medications prescribed [14]. More than half (55.4%) of the participants in our study had an education level of primary school or below, and most are taking medications for long term illness are elderly, many of whom may have limited vision and memory. Improving patients’ understanding and recall of side-effects may involve translation into an easily understood message along with an effective “patient-friendly” design [15], for example, printed in bigger fonts, avoid using medical jargon and use more graphics instead of too many words. Patients can then refer to the medication information easily at home and family members and caregivers can also understand what they need to pay attention to when managing the patients’ medications.

Engagement of community pharmacists may provide an additional manpower source to ease the burden of hospital and outpatient clinic pharmacists. A meta-analysis which looked at the impact of a pharmacist-led medication review in hospital and community settings, found that medication counseling, advice on adherence and in checking patients’ understanding of drug benefits and adverse events could improve patients’ medication knowledge and adherence [16]. The government can establish public-private partnership (PPP) with community pharmacies / pharmacists to encourage patients to receive counseling and monitoring of adherence and side effects of medication in the community. The National Health Services (NHS) in the UK has developed a New Medicine Service in collaboration with community pharmacies in order to provide early support to patients to maximize the benefits of the medication and to improve patients’ adherence to their medicines [17]. The community pharmacists are responsible to manage medication related issues and minor ailments, provide healthy living advice, and disease management support in the pharmacy setting. The Federal Government of Australia has also a partnership with approximately 5000 pharmacies to distribute medicines and other services to the Australian public [18]. Similar PPP medication therapy management program has also been developed in the US to assist patients with HIV to improve adherence to medication treatment regimens [19]. In Hong Kong, community pharmacists have limited roles and patients are generally have low acceptance level with them [20], however, it is agreed that they are competent to support patients’ self-care and manage medication-related problems in community settings [21]. There is much scope to enhance their role in improving medication knowledge particularly with the elderly in the community.

### Limitation

The response rate of the study was around 51%. It is possible that those respondents willing to be interviewed were more likely to be knowledgeable about their medications and/or more complaint with their medication, causing potential self selection bias in the study. As a result of the selection bias, the knowledge score in this population may be higher than in an average population. The length of time that the patient had been taking certain medications could also be an important factor as patients taking their medications for a longer period of time are likely to be more familiar with side-effects. It would have been useful to consider this in the initial study design.

## Conclusions

This study provides important information on the medication knowledge of patients in both primary care and specialist care clinic in Hong Kong and can help healthcare professionals and policy makers in understanding the gap of services relating to medication knowledge, compliance and safety. Lack of medication knowledge is a known and important factor contributing to medication compliance and this study was able to identify poor knowledge of medication side-effects. More importantly, this study showed substantial gaps in medication knowledge immediately following the doctor’s consultation and collection of prescribed medication from the pharmacy. People who were prescribed multiple medications and attained an education level of primary school or below appear most at risk of poor knowledge of medication side-effects. The provision of medication information to patients and the development of a public private partnership with private primary care doctors or community pharmacists to assist in medication counseling and monitoring could be a possible way to improve medication knowledge of patients with chronic conditions.

## Competing interests

The authors declare that they have no competing interests.

## Authors’ contributions

FWKC and FYYW drafted the manuscripts and performed data analysis. FWKC, WYS, CKMW and KK verified the side-effects / precautions of the medications. FWKC, FYYW and CKMW wrote the final manuscript. All authors participated in the design of the study, interpreted the results and approved the final manuscript.

## Pre-publication history

The pre-publication history for this paper can be accessed here:

http://www.biomedcentral.com/1471-2318/13/59/prepub
